# A Selectable and Excisable Marker System for the Rapid Creation of Recombinant Poxviruses

**DOI:** 10.1371/journal.pone.0024643

**Published:** 2011-09-08

**Authors:** Julia L. Rintoul, Jiahu Wang, Don B. Gammon, Nicholas J. van Buuren, Kenneth Garson, Karen Jardine, Michele Barry, David H. Evans, John C. Bell

**Affiliations:** 1 Department of Biochemistry, Microbiology and Immunology, Faculty of Medicine, University of Ottawa, Ottawa, Canada; 2 Centre for Cancer Therapeutics, Ottawa Hospital Research Institute, Ottawa, Canada; 3 Department of Medical Microbiology and Immunology, Faculty of Medicine and Dentistry, Li Ka Shing Institute of Virology, University of Alberta, Edmonton, Canada; Southern Illinois University School of Medicine, United States of America

## Abstract

**Background:**

Genetic manipulation of poxvirus genomes through attenuation, or insertion of therapeutic genes has led to a number of vector candidates for the treatment of a variety of human diseases. The development of recombinant poxviruses often involves the genomic insertion of a selectable marker for purification and selection purposes. The use of marker genes however inevitably results in a vector that contains unwanted genetic information of no therapeutic value.

**Methodology/Principal Findings:**

Here we describe an improved strategy that allows for the creation of marker-free recombinant poxviruses of any species. The Selectable and Excisable Marker (SEM) system incorporates a unique fusion marker gene for the efficient selection of poxvirus recombinants and the Cre/loxP system to facilitate the subsequent removal of the marker. We have defined and characterized this new methodological tool by insertion of a foreign gene into vaccinia virus, with the subsequent removal of the selectable marker. We then analyzed the importance of loxP orientation during Cre recombination, and show that the SEM system can be used to introduce site-specific deletions or inversions into the viral genome. Finally, we demonstrate that the SEM strategy is amenable to other poxviruses, as demonstrated here with the creation of an ectromelia virus recombinant lacking the *EVM002* gene.

**Conclusion/Significance:**

The system described here thus provides a faster, simpler and more efficient means to create clinic-ready recombinant poxviruses for therapeutic gene therapy applications.

## Introduction

Poxviruses comprise a large family of double-stranded DNA viruses that infect a wide range of hosts. Vaccinia virus (VV) is the prototypic member of the *Orthopoxvirus* genus and the best-studied virus in the poxvirus family. Since the eradication of smallpox [1], VV and other poxvirus species have continued to be used for the treatment of human disease [2], [3] in part because a greater understanding of poxvirus biology has led to safer and more efficacious poxvirus-based therapeutics. The poxvirus genome is easily genetically modified and can accommodate inserts exceeding 25 kb [4] using strategies that are dependent upon virus-encoded homologous recombination [5], [6]. Using these approaches, recombinant VV has since proven to be valuable as a vector for gene therapy in a number of therapeutic applications [4], [7], [8], [9], [10], [11], [12], [13], [14], [15]. Similarly, other members of the poxvirus family have also been explored for their potential as viral vectors for therapeutic purposes [9], [10], [16], [17]. Genetically engineered poxviruses that express immunogens from other infectious agents have shown some promise as novel vaccines against diseases like acquired immunodeficiency syndrome [11], malaria [12], tuberculosis [18], and cancer [7], [8], [10], [13]. As a cancer vaccine, poxviruses have the potential to generate a strong anti-tumoural immune response, especially when genetically modified to express cytokines like IL-2 [14] or cell surface receptors like CD70 that are indicative of oncogenic transformation [15]. Lastly, poxviruses have been successfully engineered as oncolytic agents, offering the advantage of a strong anti-tumoural immune response combined with cancer cell-specific replication [7], [16], [17], [19], [20]. A number of these poxvirus candidates have advanced to human clinical trials [10], [11], [12], [13], [19], highlighting the therapeutic potential of poxvirus recombinants.

Poxvirus recombinants are typically produced by constructing a plasmid containing the gene(s) of interest flanked by DNA sequences homologous to the desired target locus, followed by transfection of the plasmid into VV infected cells to allow for recombination of the homologous sequences between the vector and the viral genome [21]. Using traditional approaches, the frequency of recombination is typically less than 0.1% [22], and the isolation of purified recombinant virus is tedious and time-consuming. Recombinant poxviruses are often attenuated, and have reduced growth kinetics and plaque size compared to their wild type counterparts [23]. Historically, the target site of choice has been VV thymidine kinase (Tk), but any non-essential locus can be modified or disrupted in this manner. Recombinants are then isolated and plaque purified. A number of selection methods have been described including selection for Tk-positive or negative phenotypes [21], and resistance to neomycin [24] or mycophenolic acid (MPA) [25]. One can also use plaque assays to identify viruses encoding β-galactosidase [26], β-glucuronidase [27], or fluorescent reporter constructs [28].

Although these methods work well and greatly facilitate the recovery of recombinant viruses, the use of selectable markers inevitably results in the creation of a product that contains genetic information with no therapeutic value. Recombinant poxvirus therapeutics would be considered safer vectors (most notably in the view of regulatory agencies), if the selectable markers were removed from the poxvirus genome [29]. Furthermore, the expression of marker genes from recombinant poxviruses may affect the overall fitness of the virus. Demmin *et al.* have shown that the expression levels of neighboring genes can be affected by the highly active transcription of marker genes incorporated into other large DNA viruses [30].

To facilitate the removal of selectable markers, Falkner and colleagues [31], [32] developed transient selection methods wherein a selectable marker is flanked by tandem DNA repeats. The virus is stable while under selection, however the marker is lost through VV dependent homologous recombination once the selection pressure is removed. A similar system was employed by Alejo *et al*., to create ECTV recombinants [33]. Although these authors describe efficiencies in excess of 90% for removal of the selectable marker, the recombination reaction is a random and lengthy process that relies on poxvirus machinery, and involves more than six rounds of purification. Typically, the efficiency of poxvirus recombination is quite low [22], recombinant viruses are often attenuated and hard to propagate in the presence of wild-type virus [34], and many time-consuming rounds of plaque purification are needed to isolate the desired final viral product. An improved technology is needed that allows for the specific, controlled and efficient removal of selectable markers from recombinant poxvirus genomes.

Here we define a new methodological tool for rapidly producing marker-free recombinant poxviruses. This improved vector development system, which we have termed Selectable and Excisable Marker (SEM), takes advantage of the well-characterized Cre/loxP site-specific recombination system to efficiently excise the reporter gene [35], [36]. This strategy avoids the many rounds of passage that are required when using virus recombination systems to excise genes flanked by tandemly duplicated elements [31], [32], [33]. The SEM system offers the convenience of positive selection of recombinants using both fluorescent and/or drug-based strategies. Since recombinant poxvirus therapeutics are often created with multiple gene knockouts (or knock-ins), the SEM system was designed to be re-usable, therefore eliminating the use of additional reporter genes that would otherwise complicate and lengthen the overall cloning and selection processes. To demonstrate the efficiency and utility of the method, we have applied the SEM strategy to generate viruses with targeted disruptions of different genes using two different poxvirus species. Our results suggest that the SEM vector development system will not only be useful for the creation of novel poxvirus therapeutics, but also for basic virological studies.

## Results

### Characterization of the components of the SEM system

**Figure 1:**
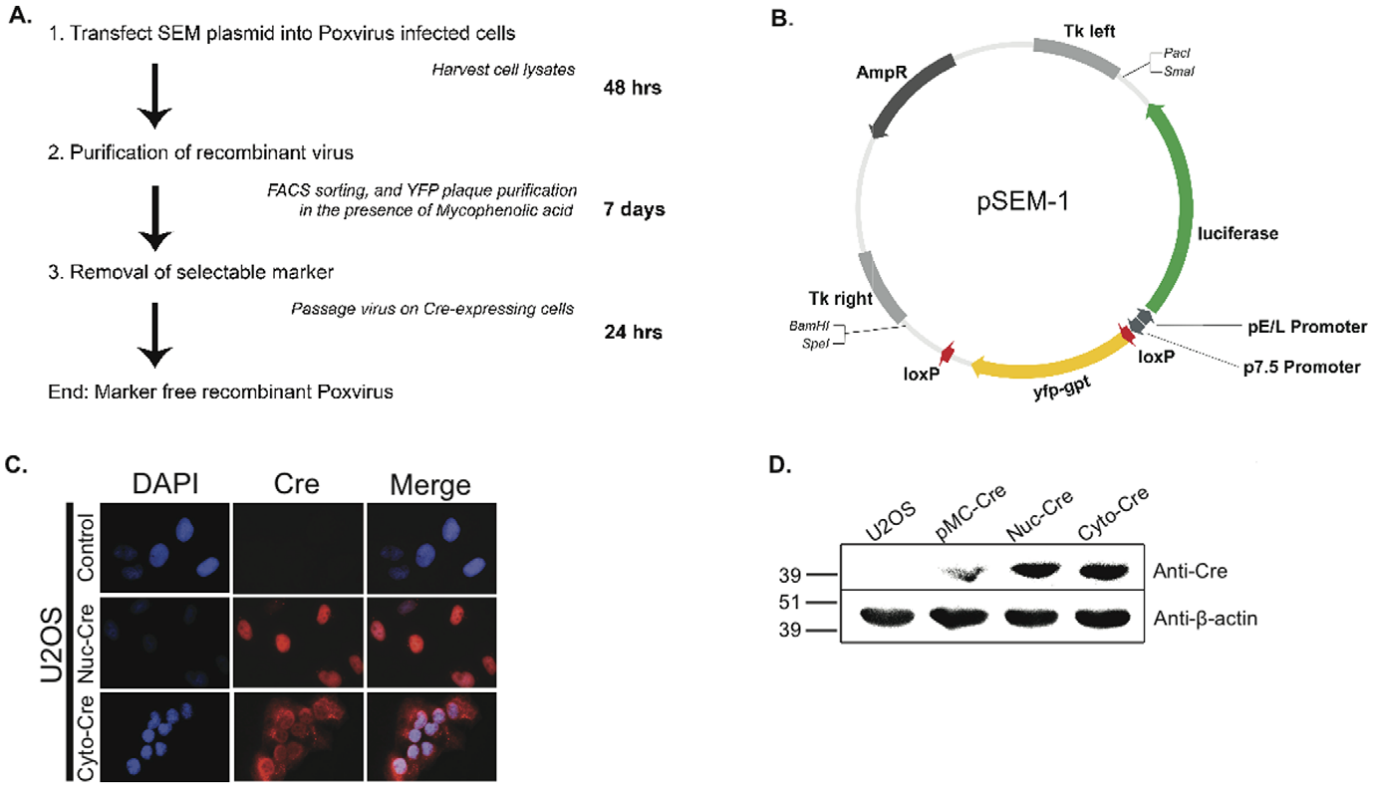
Overview of the poxvirus Selectable and Excisable Marker cloning system. (**A**) Schematic illustrating the Selectable and Excisable Marker poxvirus cloning system. (**B**) First generation poxvirus cloning vector pSEM-1 with labeled open reading frames. (**C**) Immunofluorescence detection of Cre recombinase from U2OS cell lines expressing nuclear or cytoplasmic Cre, or control U2OS cells. (**D**) Western blot analysis of Cre recombinase from U2OS cells mock transfected (U2OS), transiently transfected (pMC-Cre) or stably expressing Cre recombinase targeted to either the nucleus or the cytoplasm (Nuc-Cre and Cyto-Cre respectively).

The Selectable and Excisable Marker system is summarized in **Fig. 1A**. The first generation transfer vector pSEM-1 encoded a foreign gene (firefly luciferase), a selectable marker as a fusion between *yellow fluorescent protein* (*yfp*) and *guanine phosphoribosyltransferase* (*gpt*) genes, and loxP sites in the same orientation flanking the selectable marker, with the target insertion site as the VV Tk locus (**Fig. 1B**). To confirm that the expression of YFP was not disrupted from the creation of the YFP-GPT fusion protein, YFP expression was analyzed by western blot from U2OS cells either mock-transfected (lane 1), or transiently transfected with either pEYFP-C1 as a positive control (lane 2) or plasmids containing the *yfp-gpt* fused gene (pEYFP-gpt or pEYFP-gpt-1loxP) in lanes 3 and 4, respectively (**Figure S1A**). The YFP and YFP-GPT fusion proteins have predicted molecular weights of 26 and 45.5 kDa respectively.

The cellular distribution of Cre from Cre recombinase-expressing cell lines was analyzed by immunofluorescence from parental U2OS cells (control), nuclear Cre cells (Nuc-Cre), and cytoplasmic Cre (Cyto-Cre) cells illustrating that the absence of the nuclear localization sequence in the Nuc-Cre cells leads to accumulation of the enzyme in the cytoplasm (**Fig. 1C**). Cre expression levels were compared among mock-transfected U2OS, U2OS cells transiently transfected with pMC-Cre, and both stable cell lines (**Fig. 1D**). In cells that were transiently or stably expressing Cre-recombinase, anti-Cre western blot analysis identified a band at 35 kDa, corresponding to the Cre enzyme. The expression of Cre in the stable cell lines was 2-fold greater when compared to the transiently transfected cells.

### Isolation of a marker-free Tk-deleted, luciferase-expressing VV using the SEM system

**Figure 2:**
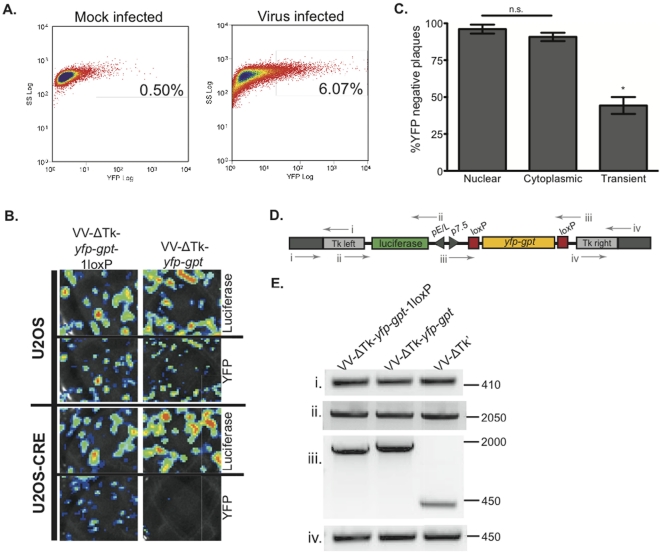
FACS purification of recombinant VV-ΔTk infected cells and Cre-recombinase mediated removal of the selectable marker. (**A**) Dot plot of YFP fluorescence versus side scatter from Fluorescence Activated Cell Sorting (FACS) analysis of U2OS cells mock infected, or infected with a mixture of parental VV and recombinant VV virus expressing YFP. (**B**) Purified recombinant VV-ΔTk-*yfp-gpt*-1loxP (control virus) or VV-ΔTk-*yfp-gpt* virus were used to infect stable cytoplasmic Cre-expressing cells (U2OS-Cre) or parental U2OS cells in 6-well plates, and were monitored for foreign gene expression (firefly luciferase) and marker gene expression (YFP-GPT fusion protein). (**C**) Percent YFP-negative VV-ΔTk' plaques on U2OS cells after passage of virus on Cre cells (nuclear or cytoplasmic stable cell lines or transiently transfected U2OS cells). (**D**) Map of the pSEM-1 plasmid indicating the primer pairs used in the PCR reactions to characterize the genome of recombinant VV-ΔTk viruses shown in panel **E**. (**E**) PCR analysis of DNA extracted from VV-ΔTk*-yfp-*gpt-1loxP (control virus), VV-ΔTk*-yfp-gpt* and VV-ΔTk' viruses. The PCR products span: **i**. across the left Tk flanking region, **ii**. across the luciferase gene, **iii**. across the *yfp-gpt* selectable marker, **iv**. across the right the Tk flanking region. n.s = not-significant, * p = 0.001.

(YFP)-based fluorescence activated cell sorting (FACS) was performed on mock infected U2OS cells as a control, and U2OS cells that had been infected with a mixture of parental VV (Wyeth strain), and recombinant VV generated from pSEM-1 expressing the YFP-GPT fusion protein (VV-ΔTk-*yfp-gpt*) (**Fig. 2A**). To ensure the highest possible FACS stringency, a cut-off of 0.5% background fluorescence was maintained by comparing the mock-infected U2OS cells to the recombinant YFP-expressing VV infected U2OS cells (235 positive background cells from 47,421 U2OS cells counted, compared to 3004 positive recombinant VV infected cells, from 49,498 U2OS counted). These sorted cells were then mixed with uninfected U2OS cells, plated into multi-well dishes, and subjected to two more rounds of YFP^+^ plaque purification.

To promote fast and simple removal of the *yfp-gpt* cassette from recombinant viruses generated using the SEM system, viruses were passaged on a U2OS cell line expressing a cytoplasmic form of Cre recombinase. VV-ΔTk-*yfp-*gpt-1loxP (control virus) and VV-ΔTk*-yfp-gpt* recombinant VV were passaged on either parental U2OS cells, or U2OS cells stably expressing cytoplasmic Cre recombinase (U2OS-Cre) (**Fig. 2B**). The U2OS cells were monitored for both YFP fluorescence and for luciferase-mediated bioluminescence using the IVIS Imager (Xenogen) and Living Image® v2.5 software. As shown in the top half of **Fig. 2B**, both VV-ΔTk-*yfp-gpt*-1loxP control virus, and VV-ΔTk-*yfp-gpt* viruses express luciferase and YFP when used to infect parental U2OS cells. Infection of U2OS-Cre cells by the VV-ΔTk*-yfp-gpt*-1loxP and VV-ΔTk-*yfp-gpt* viruses led to strong luciferase transgene expression, however expression of YFP is only detectable from cells infected with the VV-ΔTk-*yfp-gpt*-1loxP virus. This illustrates the qualitative efficiency by which Cre-expressing U2OS cells excise loxP-flanked markers, as well as the stability of the transgene expression during the recombination reaction. To quantitatively determine the efficiency of the Cre recombination reaction, U2OS cells expressing Cre recombinase were infected with the VV-ΔTk*-yfp-gpt* virus. Virus progeny was then analyzed for the percent YFP positive versus negative virus plaques by U2OS plaque assay. As seen in Figure 2C, both cytoplasmic and nuclear Cre expression from stable cell lines resulted in nearly 100% efficiency for marker gene excision, whereas transient transfection of Cre recombinase was only 44% efficient.

The genomic composition of the VV recombinant viruses was analyzed by PCR of viral DNA before and after removal of the *yfp-gpt* cassette. A schematic of the virus genome at the Tk insertion site is shown (**Fig. 2D**) to illustrate the primer pairs used in the analysis. Primers were designed to amplify regions of DNA at the insertion site (i and iv), surrounding the luciferase transgene (ii), and across the *yfp-gpt* selectable marker (iii). For three of the PCR reactions (primer pairs i, ii, and iv), the amplicons for each of the viruses tested were the same and show that the Cre recombination reaction does not affect the genome structure outside of the 2loxP region (ii), and that the Tk locus was the site of homologous recombination (i, iv) (**Fig. 2E**). Prior to passage on Cre-expressing cells, PCR using primer pair (iii) of DNA from both VV-ΔTk-*yfp-gpt*-1loxP and VV-ΔTk-*yfp-gpt* viruses produced bands at 1900 and 2000 bp, respectively. The 100 bp difference can be attributed to the lack of one loxP site in the VV-ΔTk-*yfp-gpt*-1loxP virus. PCR of DNA from the marker-free recombinant VV-ΔTk' virus using primer pair (iii), produced a much smaller band due to Cre-mediated deletion of the *yfp-gpt* gene.

### Genomic analysis and confirmation of identity of 3 independent clones of the VV-ΔTk-*yfp-gpt* and VV-ΔTk' viruses

**Figure 3:**
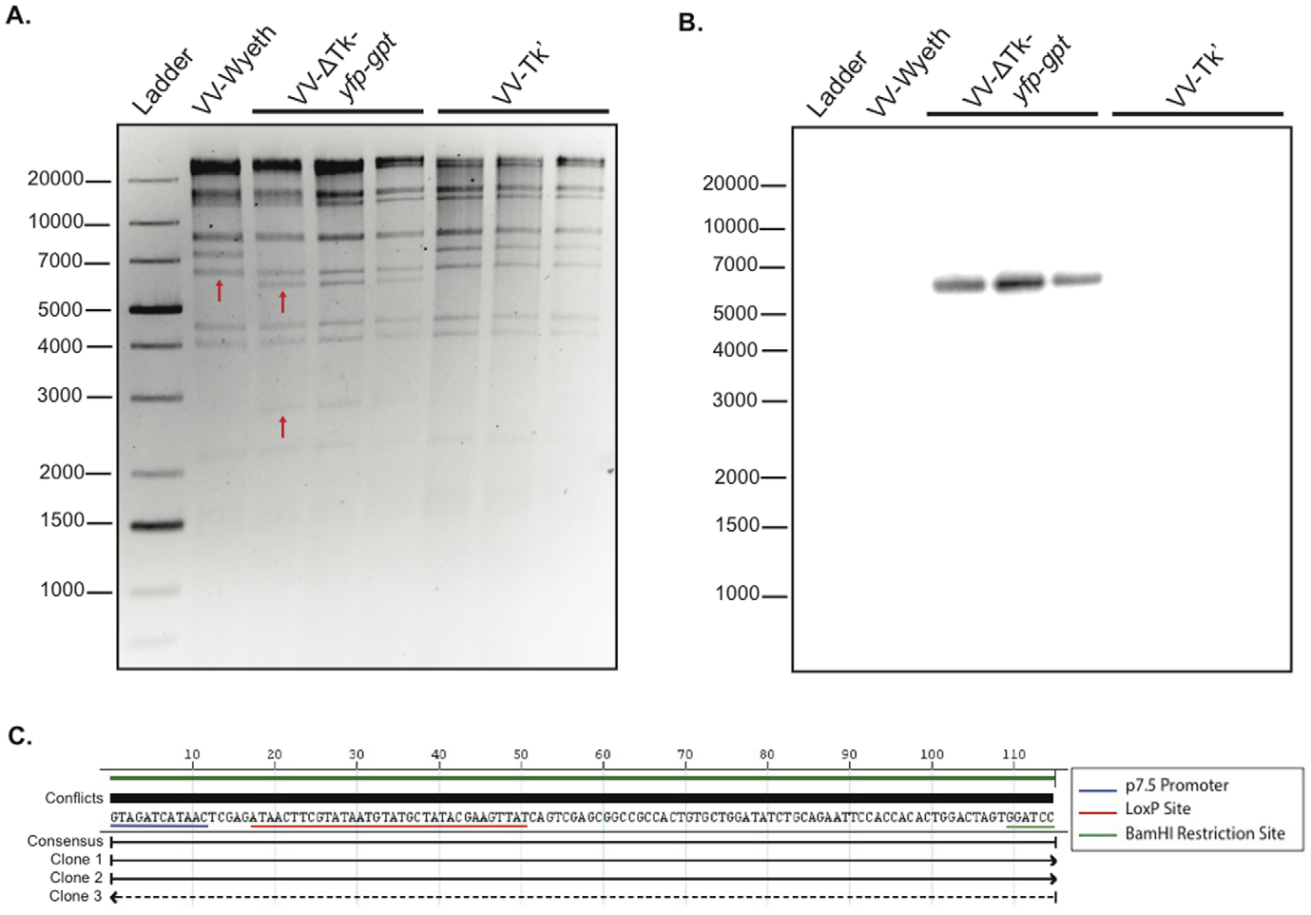
Confirmation of genomic composition of 3 independent recombinant VV-ΔTk viruses. (**A**) An ethidium bromide stained DNA gel of genomic HindIII restriction digests of viral DNA isolated from parental VV (Wyeth Strain), 3 clones of VV-ΔTk-*yfp-gpt* and 3 clones of VV-ΔTk'. Arrows indicate the Tk insertion site (VV-Wyeth), and the unique bands that result from insertion of the *yfp-gpt* cassette (VV-ΔTk-*yfp-gpt*). (**B**) Southern hybridization of the DNA gel in **A** identifying the *yfp* insert present in the genome of the VV-ΔTk-*yfp-gpt* clones, but not in parental VV-Wyeth or the VV-ΔTk' clones. (**C**) DNAStar sequence alignment at the *yfp-gpt* insertion site of DNA isolated from the 3 VV-ΔTk' clones post Cre passage.

The genomic composition of the VV-ΔTk-*yfp-gpt* and VV-ΔTk' viruses was analyzed by restriction enzyme digestion, southern blot hybridization and sequencing analysis at the Cre-recombination site (**Fig. 3**). Three independent clones of the VV-ΔTk-*yfp-gpt* virus were compared to the parental VV Wyeth strain, before and after passage on Cre-expressing cells. The DNA fragment containing the Tk insertion site is highlighted (arrow) in the digest of the parental VV Wyeth virus. Interestingly, the *yfp-gpt* cassette included a HindIII restriction site. This led to a unique DNA digest for the VV-ΔTk-*yfp-gpt* clones (**Fig. 3A**). The DNA fragment containing the Tk insertion site is disrupted in digests of the VV-ΔTk-*yfp-gpt* viruses, and is represented by unique bands at ∼6000 and ∼2800 bp (see arrows, **Fig. 3A**). Digests of the VV-ΔTk' clones resemble the parental VV digest, since the *yfp-gpt* cassette was removed by Cre-recombination. Importantly, the southern hybridization for *yfp* demonstrates that there was only 1 insertion site of *yfp-gpt* during poxvirus homologous recombination (**Fig. 3B**). DNA sequence analysis of the 3 VV-ΔTk' clones was performed to illustrate the consistency of the residual DNA signature following Cre-recombination. A DNAStar sequence alignment of the DNA from each of the 3 VV-ΔTk' clones revealed a consensus sequence with no conflicts (**Fig. 3C**). As predicted, the residual DNA signature contained 1 loxP site and remnants from the pSEM-1 vector.

### Construction of the second generation SEM cloning vector and the VV-Δ*I4L* viruses

**Figure 4:**
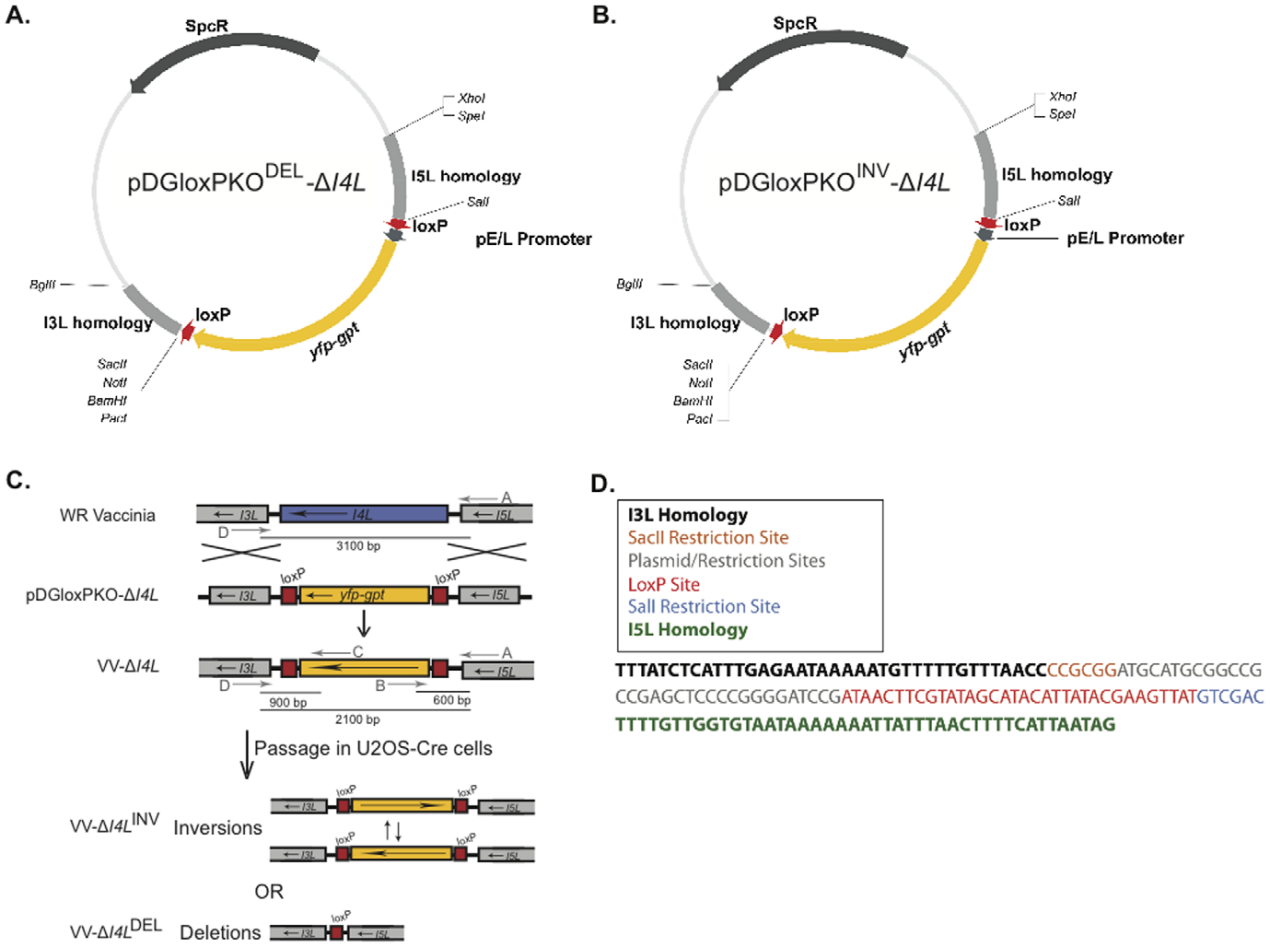
Creation of the VV-Δ*I4L* mutants from the second generation SEM cloning vectors. (**A**) Map of cloning vector pDGloxPKO^DEL^-Δ*I4L* and (**B**) pDGloxPKO^INV^-Δ*I4L* with labeled open reading frames. (**C**) Schematic displaying the strategy for knock-out of the *I4L* open reading frame from VV strain WR, and possible outcomes of Cre-recombination of recombinant VV-Δ*I4L* virus generated from either the pDGloxPKO^DEL^-Δ*I4L* or pDGloxPKO^INV^-Δ*I4L* vectors. (**D**) DNA sequence analysis of the VV-Δ*I4L* ^DEL^ virus post Cre passage.

The second generation SEM plasmid, termed pDGloxPKO was designed with multiple cloning sites flanking the *yfp*-*gpt* cassette. This permits insertion of homologous targeting sequences flanking the selectable marker and/or therapeutic transgene. This vector was also designed such that the *yfp-gpt* cassette and its early/late viral promoter are flanked by loxP sites. To test the importance of loxP site orientation during Cre-recombination, two pDGloxPKO vectors were created with the loxP sites in either the same orientation (pDGloxPKO^DEL^) (**Figure S2A**), or oriented towards each other (pDGloxPKO^INV^) (**Figure S2B**). Inserting homologous sequences of DNA from the VV genome flanking the *I4L* gene (I3L and I5L homology) into both pDGloxPKO^DEL^ and pDGloxPKO^INV^ vectors generated pDGloxPKO^DEL^-Δ*I4L* and pDGloxPKO^INV^-Δ*I4L*, respectively (**Fig. 4A and 4B**). These vectors were used to create two strains of recombinant VV in which the *I4L* locus is disrupted by vector-derived *yfp-gpt* cassette sequences (see schematic in **Fig. 4C**). Using vector pDGloxPKO^DEL^-Δ*I4L*, which contains identically oriented loxP sites flanking the *yfp-gpt* cassette led to the generation of strain VV-Δ*I4L* ^DEL^, whereas using vector pDGloxPKO^INV^-Δ*I4L* in which the inserted *yfp-gpt* cassette is flanked by loxP sites oriented towards each other generated virus VV-Δ*I4L* ^INV^. The genomic composition of the VV-Δ*I4L* ^DEL^ virus post Cre passage was analyzed by sequencing at the excision site of *yfp-gpt* and revealed the Cre/loxP signature remaining in the viral genome post Cre-recombination (**Fig. 4D**). As expected, the virus contains 1 loxP site, and remnants from the pDGloxPKO vector. To exclude the possibility that there were multiple *yfp-gpt* insertion sites, the entire genome of VV-Δ*I4L* ^DEL^ virus post Cre passage was sequenced. The sequence of the VV-Δ*I4L* ^DEL^ recombinant was compared to the sequence of the parental VV (Western Reserve) using a dotplot analysis (**Figure S3A**). These data illustrate that the VV-Δ*I4L* ^DEL^ virus is disrupted only at the *I4L* locus, thereby confirming 1 insertion site at the desired locus.

### Cre-mediated recombination of viral DNA is dependent upon loxP site orientation

**Figure 5:**
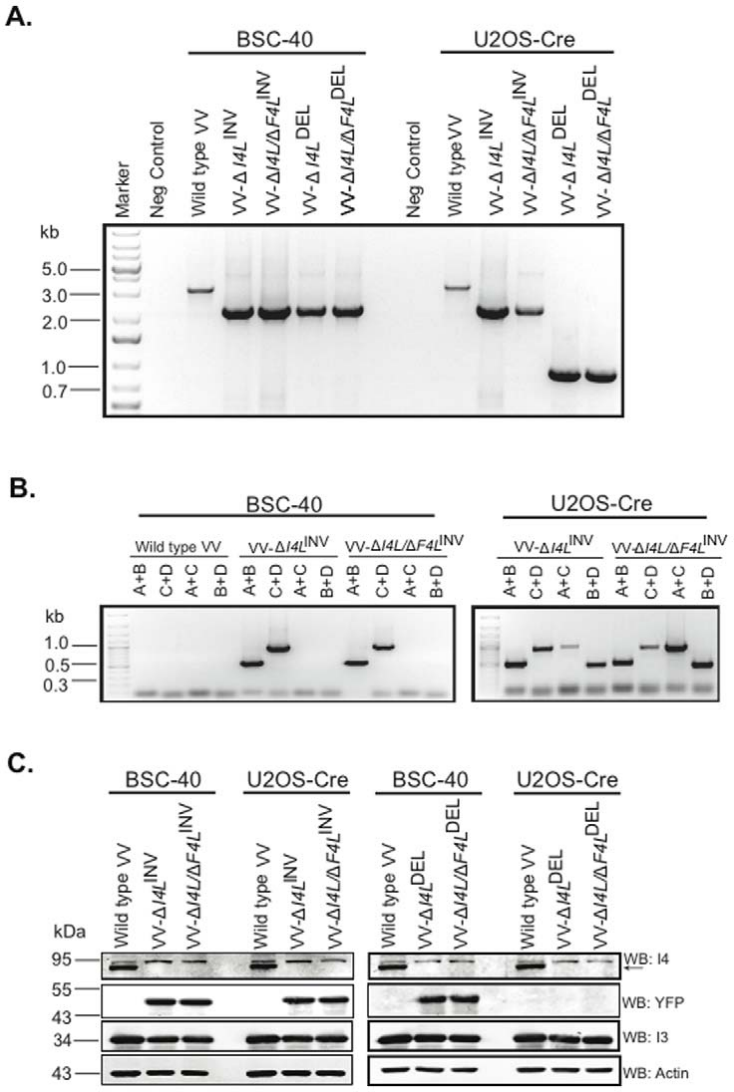
Cre-mediated recombination of vaccinia virus DNA is dependent upon loxP site orientation. (**A**) PCR analysis of the *I4L* locus using primers flanking the *yfp-gpt* cassette of two purified Δ*I4L* strains generated from either the pDGloxPKO^DEL^-Δ*I4L* or pDGloxPKO^INV^-Δ*I4L* vectors before (BSC-40) and after (U2OS-Cre) Cre-recombination. (B) PCR analysis of two purified Δ*I4L* strains generated from the pDGloxPKO^INV^-Δ*I4L* vector with primers amplifying inside and outside the *yfp-gpt* cassette before (BSC-40) and after (U20S-Cre) Cre-recombination. (C) Western blot analysis of two purified Δ*I4L* strains generated from either the pDGloxPKO^DEL^-Δ*I4L* or pDGloxPKO^INV^-Δ*I4L* vectors for I4, YFP, I3 (positive control for infection) and actin before (BSC-40) and after (U20S-Cre) Cre-recombination.

Both the pDGloxPKO^DEL^ and pDGloxPKO^INV^ plasmids were used to explore the dependence of loxP orientation on Cre-mediated recombination of poxvirus genomes. Previous work has shown that Cre-mediated recombination between identically-oriented loxP sites generates deletions while oppositely oriented loxP sites lead to inversion of DNA sequences [35], [36], but it remained to be formally proven that this would also hold-true in virus-infected cells. Recombinant Δ*I4L* viruses were analyzed by PCR for the presence of the *I4L* gene before (BSC-40 passage) and after (U2OS-Cre passage) Cre recombination (**Fig. 5A**). As an additional test of the SEM approach, we have included *F4L*-inactivated recombinants in these experiments, as Δ*F4L* virus backgrounds have been reported to have severe growth kinetics compared to their wild-type counterpart [23]. Referring to the expected amplicon sizes and primer pairs illustrated in **Fig. 4C**, the PCR products generated using primers flanking the *yfp-gpt* cassette (A+D) exhibited sizes indicative of replacement of the *I4L* gene with the *yfp-gpt* cassette in viruses cultured in BSC-40 cells (**Fig. 5A**, left panel). Upon passage in Cre-expressing cells, only those viruses produced using pDGloxPKO^DEL^ vectors (Δ*I4L* ^DEL^, and Δ*I4L/*Δ*F4L* ^DEL^) exhibited the deletion of the *yfp-gpt* cassette (**Fig. 5A**, right panel). This shows that Cre-mediated deletions only occur when the DNA is flanked by identically oriented loxP sites.

The orientation of the *yfp-gpt* cassette in recombinant viruses produced using the pDGloxPKO^INV^-Δ*I4L* vector were also analyzed by PCR before and after passage through Cre-expressing cells. We used primer pairs designed to amplify the *yfp-gpt* cassette in the forward orientation (pairs A+B, C+D), and also in the case of *yfp-gpt* inversions (pairs A+C, B+D). The viruses isolated from BSC-40 cells produced PCR products consistent with a single orientation identical to that seen in original plasmid (**Fig. 5B**, left panel). However, upon passage through Cre-expressing cells, the PCR products displayed a pattern characteristic of a mix of two different arrangements of loxP flanked inserts (**Fig. 5B**, right panel).

Western blot analysis was used to confirm deletion of the *I4L* locus of all Δ*I4L* strains. Recombinant Δ*I4L* viruses produced with either pDGloxPKO^INV^-Δ*I4L* or pDGloxPKO^DEL^-Δ*I4L* vectors led to inactivation of I4 protein expression and this inactivation was specific since I3, (expressed from the neighbouring gene, *I3L*) levels remained unchanged (**Fig. 5C**). The YFP-GPT protein was only deleted from strains that had been generated with the pDGloxPKO^DEL^-Δ*I4L* targeting vector *and* passaged in Cre-expressing cells (**Fig. 5C**, right panel), further confirming that deletion events need both identically-oriented loxP sites and exposure to Cre activity. Collectively, these results demonstrate that the SEM vector system can be used to either delete or invert sequences within the viral genome, upon passage of the selected strains in Cre-expressing cells.

### The SEM system can be used with other members of the Poxvirus family

**Figure 6:**
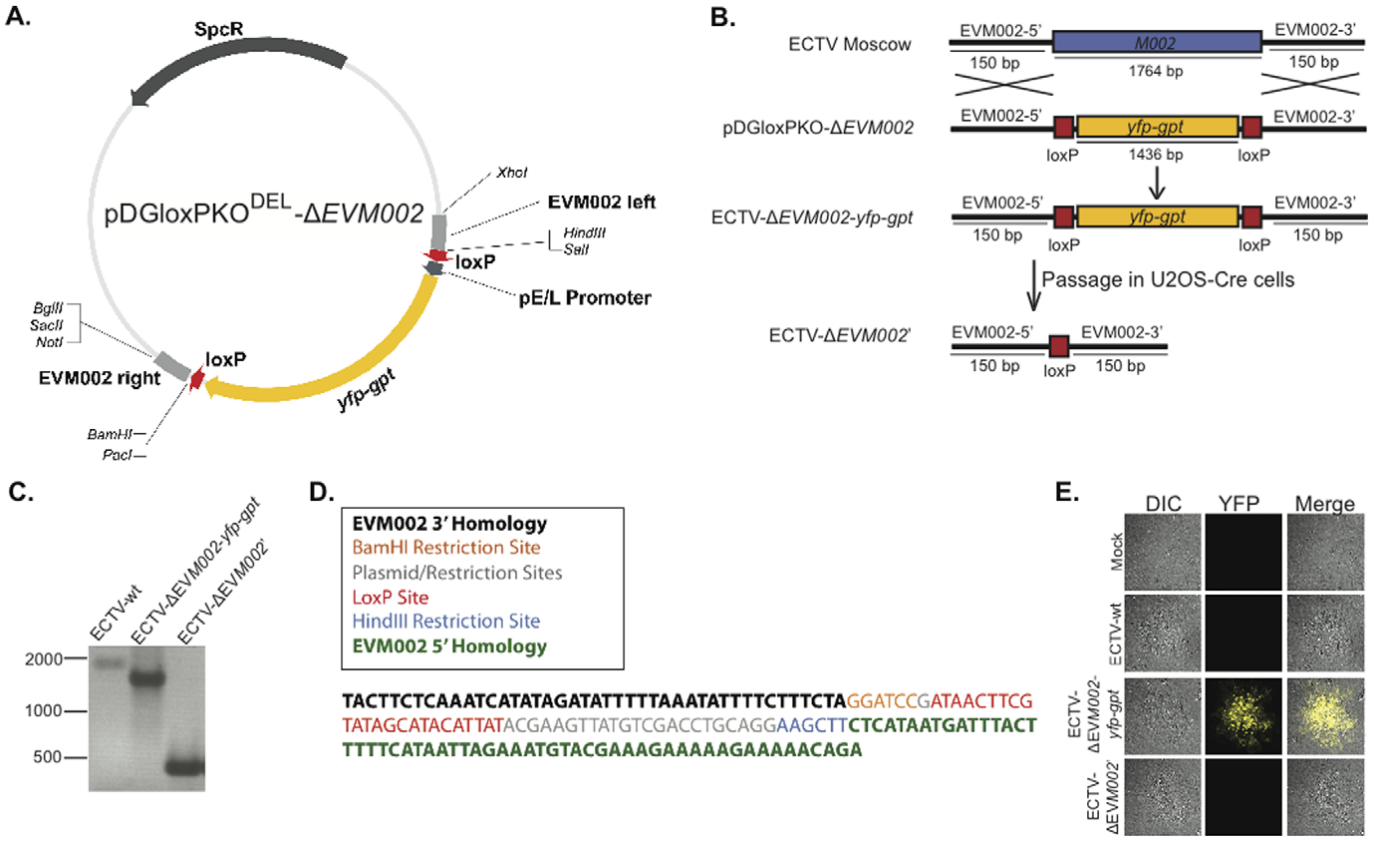
Generation of a recombinant ECTV using SEM. (**A**) Map of pDGloxPKO^DEL^-Δ*EVM002* with labeled open reading frames. (**B**) Schematic displaying the strategy for knock-out of the *EVM002* open reading frame from ECTV. (**C**) PCR analysis of viral DNA. Primers were used to amplify the regions of pDGloxPKO-Δ*EVM002* homology from wild-type ECTV (ECTV-wt), ECTV-Δ*EVM002*-*yfp-gpt*, and ECTV-Δ*EVM002'*. (**D**) DNA sequence analysis of the ECTV-Δ*EVM002*' virus (post Cre passage). (**E**) Confocal microscopy was used to detect YFP fluorescence in mock infected BGMK cells or BGMK cells infected with ECTV-wt, ECTV-Δ*EVM002*-*yfp-gpt*, or ECTV-Δ*EVM002*'. A 40× magnification lens was used to detect differential interference contrast (DIC), and YFP fluorescence.

The SEM system was also used to create a recombinant ECTV (strain Moscow) lacking the *EVM002* gene using the transfer plasmid pDGloxPKO^DEL^-Δ*EVM002* (**Fig. 6A**). Referring to the schematic outlined in **Fig. 6B**, ECTV viral recombination with pDGloxPKO^DEL^-Δ*EVM002* with subsequent MPA drug selection created the primary recombinant ECTV-Δ*EVM002*-*yfp-gpt*. The virus was passaged through U2OS-Cre cells to obtain the marker-free recombinant virus ECTV-Δ*EVM002*'. The genomic composition of wild type ECTV (ECTV-wt), ECTV-Δ*EVM002*-*yfp-gpt*, and ECTV-Δ*EVM002*' viruses was confirmed by PCR analysis (**Fig. 6C**). These PCR analyses demonstrate at the genomic level the deletion of the *EVM002* gene, the insertion of the *yfp-gpt* cassette, and its subsequent removal following Cre-mediated recombination. The genomic composition of the ECTV-Δ*EVM002*' was analyzed by sequencing at the excision site of *yfp-gpt* to illustrate the Cre/loxP signature remaining in the viral genome post recombination (**Fig. 6D**). As expected, the virus contains 1 loxP site, and remnants from the pDGloxPKO vector. YFP protein expression from the ECTV viruses was also analyzed by confocal microscopy (**Fig. 6E**). Mock-infected BGMK cells were compared to ECTV-wt, ECTV-Δ*EVM002*-*yfp-gpt*, and ECTV-Δ*EVM002*' infected cells for YFP fluorescence. Only those cells that were infected with recombinant ECTV-Δ*EVM002*-*yfp-gpt* expressed detectable levels of YFP fluorescence.

## Discussion

Poxviruses have been, and will continue to be, important therapeutics for the prevention and treatment of human diseases [2], [3], [10], [11], [12], [13], [19]. Arguably, the application of VV for the eradication of smallpox has been one of the most important medical advances in human history. A variety of poxvirus based vaccine vectors have been developed for both human and veterinary infectious diseases and more recently as agents for the treatment of cancer [2], [7], [8], [10], [13], [14], [15], [16], [17], [19], [20]. During therapeutic development it is desirable to use marker genes for both construction of novel vectors but also for pre-clinical and even early phase clinical experimentation. Unfortunately as products mature, there becomes a transition point where marker genes are no longer necessary and, in the view of regulatory agencies, may even compromise the safety of the vector [29].

The SEM system provides a faster, simplified and more efficient means to create marker-free recombinant poxviruses [31], [32], [35]. As outlined in **Fig. 1A**, the SEM cloning strategy can be summarized in three basic steps: transfection of poxvirus-infected cells with an SEM vector, purification of resulting recombinants through FACS and/or drug selection, and finally removal of the selectable marker by virus passage in Cre-expressing cells. The first generation pSEM-1 vector (**Fig. 1B**) was created to demonstrate the “proof of principle” that it was possible to create marker-free recombinant poxviruses with our system, while stably retaining a functional transgene (in our case luciferase) during the Cre-recombination reaction (**Fig. 2B**). The second generation SEM plasmids (**Figure S2**) broaden the applicability of the SEM system to other poxvirus loci, and other poxvirus genera by the insertion of two multiple cloning sites (MCS) flanking the *yfp-gpt* cassette and loxP sites. Sequences homologous to any poxvirus gene can be added to the pDGloxPKO vector, as we have demonstrated with the ectromelia virus recombinant constructed from pDGloxPKO^DEL^-Δ*EVM002* (**Fig. 6**).

The most labour-intensive part of traditional poxvirus cloning strategies is the isolation and purification of desired recombinants from a heterogeneous population. To simplify the isolation of pure recombinant poxvirus clones, we have included both drug (*gpt*) and optical (*yfp*) selection strategies in the SEM system. The flexibility and simplicity of this approach ensures that the SEM system will be amenable to any research laboratory. MPA drug selection is relatively inexpensive and historically has shown to be an efficient means to purify poxvirus recombinants [25]. Indeed, we have shown here that MPA selection alone is sufficient to isolate a pure recombinant poxvirus population in as few as three passages (e.g. VV-Δ*I4L*, **Fig. 4** and ECTV-Δ*EVM002*, **Fig. 6**). We have also shown the usefulness of fluorescent sorting strategies to rapidly enrich for the recombinant virus population by subjecting the initial transfection/infection reaction to FACS analysis (**Fig. 2A**). Alternatively, a more traditional approach of using fluorescent microscopy to identify and pick individual plaques expressing YFP can be used. The flexibility of the SEM system in the purification of recombinant poxviruses is particularly important when working with highly attenuated strains. Importantly, we show here that even the double deleted Δ*I4L/*Δ*F4L* VV strains, which have severe replication defects and exhibit a small plaque phenotype [23], can be produced and purified using the SEM system.

We had hypothesized that expression of cytoplasmic Cre-recombinase would maximize recombination efficiency since poxviruses replicate in the cytoplasm. Indeed a single passage of the VV-ΔTk-*yfp-gpt* virus on U2OS cells expressing cytoplasmic Cre is sufficient to eliminate nearly all of the *yfp-gpt* cassette from the viral genome (**Fig. 2B and 2C**). However, infection of U2OS cells stably expressing *nuclear* Cre still led to nearly 100% recombination efficiency (**Fig. 2C**). Not unlike other proteins, the Cre enzyme is initially located in the cytoplasm prior to transport to the nucleus. We speculate that in cells selected for high levels of Cre expression, the amount of enzyme is not limiting for the excision reaction and thus even small amounts, or temporary cytoplasmic expression of Cre from nuclear-cre expressing cells may be sufficient to facilitate recombination. Importantly, despite lower Cre-expression levels achieved from transient transfection of U2OS cells with pMC-Cre (**Fig. 1D**), the recombination frequency was still >40% (**Fig. 2C**). These data illustrate how the SEM system can be used with any transfectable cell line while maintaining the efficiency of the Cre/loxP reaction.

To confirm that the residual DNA signature was consistent for all recombinant viruses post Cre recombination, DNA sequencing was performed at the insertion site (**Figs. 3C** **,** **4D** **,** **6D**). In all cases, one 34 base pair loxP site remains in the viral genome, with some remnants of the respective SEM cloning vectors. We have observed no obvious impact of retention of this 34 base pair sequence on virus replication and indeed this has been studied extensively in mammalian systems where the retention of loxP sequences has no impact on mRNA stability, promoter activity or genome integrity [37], [38]. To further validate the consistency of the recombination events of the SEM system, 3 clones of the VV-ΔTk virus pre (VV-ΔTk-*yfp-gpt*) and post (VV-ΔTk') Cre passage were analyzed by HindIII DNA restriction digest and southern hybridization (**Fig. 3**). As predicted, the DNA signatures are consistent, and the Tk locus is the only site of poxvirus recombination. Since the VV-Δ*I4L* and ECTV-Δ*EVM002*' recombinant viruses were purified as heterogeneous populations (MPA drug selection), the sequence analysis is representative of all recombination events. To confirm that there was only 1 site of *yfp-gpt* integration in the VV-Δ*I4L* ^DEL^ virus, the entire genome of the virus was sequenced. A dotplot comparison of recombinant VV-Δ*I4L* ^DEL^ to parental VV illustrates that the recombination event was targeted to the *I4L* locus (**Figure S3**).

Using the second generation SEM cloning vectors (pDGloxPKO^DEL^ and pDGloxPKO^INV^), we have shown for the first time that similar to recombination of cellular DNA [35], [36], Cre-mediated recombination between identically-oriented loxP sites generates deletions, while oppositely oriented loxP sites leads to inversions of viral DNA sequences (**Fig. 5**). Interestingly, targeted inversions of the *yfp-gpt* cassette when generating viruses using the pDGloxPKO^INV^-Δ*I4L* targeting vector identified an additional application for the SEM system. Site-specific inversions may be useful for transcriptional interference studies [39] in which a poxvirus promoter could drive the same mRNA from opposite strands. This approach would allow one to assess the potential strand-specific influence of neighbouring transcriptional processes and read-through transcripts on the efficiency of transcription of the reporter mRNA.

In summary, the SEM strategy provides an efficient means to “surgically” manipulate viral genomes by adding or deleting genes without leaving unwanted marker genes behind. We have demonstrated the applicability of the SEM system to create recombinant viruses in two different species, targeting three different poxvirus loci by deletions and/or insertions, including strains with severe replication defects. By inclusion of fluorescence and drug selection strategies, the SEM system offers both flexibility and simplicity for the purification of recombinant viruses. The *yfp-gpt* cassette was also shown to be useful during Cre-recombination to monitor efficiency, which could be enhanced even further by making use of a reverse *gpt* selection strategy [40]. This system will be especially useful when multiple deletions or transgenes are to be added sequentially to a single vector. Finally, the tools developed in the SEM system may also contribute to basic virology research, like those that involve transcriptional interference studies.

## Materials and Methods

### Antibodies and reagents

The following primary antibodies were used: rabbit anti-GFP/YFP (Abcam), Cre (Novagen), VV I4 (a gift from Dr. C. Mathews, Oregon State University), mouse anti-β-actin (Sigma-Aldrich) and VV I3 [41]. Secondary antibodies conjugated to horseradish peroxidase (BioRad) or infrared dyes (Li-Cor) were used to detect primary antibodies in immunoblotting. OptiMEM (Invitrogen) was used for transfection experiments.

### Cells

The following cells were purchased from American Type Tissue Collection: human osteosarcoma (U2OS), human embryonic kidney, large T antigen transformed (293T), and green monkey kidney (BSC-40) cells. Buffalo green monkey kidney (BGMK) cells were purchased from Diagnostic Hybrid. Cell lines were maintained in Dulbecco's modified Eagle's medium (Hyclone) supplemented with 10% fetal calf serum (Cansera), penicillin (100 U/ml), and streptomycin sulfate (100 µg/ml) at 37°C in 5% CO_2_. All cell lines were tested and found clear of mycoplasma contamination.

### Plasmids

Plasmids pEYFP-C1, pLPCX, and pLXSN were purchased from Clontech and pGEM-T was purchased from Promega. Plasmid pMC-Cre [42] was a gift from Dr. Klaus Rajewsky (Harvard Medical School), pSEL-eGFP [20] and pSC65-luc [43] were gifts from Dr. David Bartlett (University of Pittsburgh) and pHIT60 and pHIT456 were gifts from Dr. Ian Lorimer (University of Ottawa).

### Cloning – pSEM-1 vector

The *gpt* gene was first excised from pSEL-eGFP with *Bse*Y1, repaired, and ligated into a pT7blue-3 cloning vector (Novagen). The *gpt* gene was then excised with *Sal*I and *Bam*HI and ligated, in frame, into *Xho*I/*Bam*HI cut pEYFP-C1. This created pEYFP-gpt bearing a fusion of the two polypeptides. The first loxP sequence was constructed by annealing together two oligonucleotides with the same sequence (loxP, **Table S1**), using the In-Fusion-2 method (Clontech) [44]. The pEYFP-gpt plasmid was then cut with *Nhe*I and recombined with the double-stranded DNA loxP site to obtain ploxP-EYFP-gpt. The second loxP sequence was inserted in a similar way by cutting ploxP-EYFP-gpt at the *Bam*HI site, and inserting the double-stranded oligonucleotide loxP (**Table S1**). The resulting plasmid, p2loxP-EYFP-gpt, contained two loxP sites with the same orientation flanking the gene encoding the YFP-GPT fusion protein.

The pSEM-1 plasmid was assembled by inserting the loxP*-yfp-gpt-*loxP cassette into pSC65-luc. This was accomplished by cutting p2loxP-EYFP-gpt and pSC65-luc plasmids with *Xho*I and *Bam*HI restriction enzymes, and ligating the loxP-*yfp*-*gpt*-loxP insert into the VV Tk gene in pSC65-luc. Two vectors were created: a negative control vector containing only the first loxP site (pSEM-1ctrl), and a vector containing two loxP sites inserted in the same orientation and flanking the reporter construct (pSEM-1) (**Fig. 1B**).

### Cloning – pDGloxPKO vectors

A second series of vectors were assembled using gene synthesis (GeneArt) and contained two multiple cloning sites flanking the loxP sites. To test the importance of loxP orientation, two versions of these plasmids were created with the loxP sites either in the same (pDGloxPKO^DEL^) or in the opposite orientation (pDGloxPKO^INV^) with respect to one another (**Figure S2**).

### Cloning – pDGloxPKO-ΔI4L vectors

The PCR and primers I5L (left) plus I5L (right) (**Table S1**) were used to prepare a ∼430 bp PCR product encoding sequences flanking the “*I5L*” side of the VV *I4L* locus. Similarly the PCR and primers I3L (left) plus I3L (right) (**Table S1**) were used to produce a ∼340 bp PCR product containing sequences flanking the “*I3L*” side of the *I4L* locus. These DNAs were then cloned into the *Spe*I/*Sal*I (*I5L* homology) and *Sac*II/*Bgl*II (*I3L* homology) restriction sites of pDGloxPKO^INV^ and pDGloxPKO^DEL^. Adding these sequences created vectors pDGloxPKO^INV^-Δ*I4L* and pDGloxPKO^DEL^-Δ*I4L* (**Fig. 4**).

### Cloning – pDGloxPKODEL-ΔEVM002 vector

The PCR and primers EVM002-5′ (left) plus EVM002-5′ (right) (**Table S1**) were used to prepare a 150 bp product encoding sequences to the left of the *EVM002* gene. Similarly the PCR and primers EVM002-3′ (left) plus EVM002-3′ (right) (**Table S1**) were used to produce a 150 bp product encoding sequences to the right of the *EVM002* gene in the ectromelia virus (ECTV) genome. These two 150 bp PCR fragments were cloned into pGEM-T vectors (Promega). The fragment encoding sequences located on the left side of the *EVM002* gene was subsequently cloned into the pDGloxPKO^DEL^ vector using the *Xho*I and *Hind*III restriction sites to create pDGloxPKO-*EVM002*-5′. Finally, the EVM002-3′ 150 bp was cloned into pDGloxPKO-*EVM002*-5′ using the *Bam*HI and *Not*I restriction sites to create pDGloxPKO^DEL^-Δ*EVM002*.

### Cloning – Cre plasmids

The gene encoding a form of Cre protein lacking a nuclear localization sequence (NLS) was PCR amplified from pMC-Cre using Taq polymerase and primers NLS-free Cre (left) and NLS-free Cre (right) (**Table S1**). The PCR product was digested with *Xho*I and *Not*I and ligated into pLPCX cut with the same enzymes. This produced plasmid pLPCX-Cyto_Cre. A Cre plasmid was also created with the NLS intact by digesting Cre from pMC-Cre, and inserting the gene into the *Xho*I site of the pLXSN vector to form pLXSN-Nuc_Cre.

### U2OS-Cre cells

Retroviral transduction methods were used to produce cells expressing nuclear- or cytoplasmic-localized Cre protein. A stock of transducing particles was first produced by using calcium phosphate to co-transfect 293T cells with 7 µg of plasmid DNA (either pLPCX-Cyto_Cre or pLSXN-Nuc_Cre) along with the retroviral helper plasmids pHIT60 and pHIT456 [45]. Virus-containing supernatants were harvested two days later and the debris removed with a 0.45-µm filter. To perform the transduction experiment, 2.5×10^5^ U2OS cells were infected with 2 mL of the virus for 2 hr in the presence of 8 µg/mL of polybrene. Two days later, G418 (800 µg/mL) or puromycin (1 µg/mL) were added and drug-resistant recombinants were harvested a week later. Purified recombinant poxviruses were passaged 1-to-3 times in these cells to remove loxP-flanked genes.

### Recombinant viruses

Viruses constructed from the pSEM-1 vector were produced from the VV strain Wyeth [7]. U2OS cells were infected with VV at a multiplicity of infection (MOI) of 0.01 and then transfected with plasmid pSEM-1 or pSEM-1ctrl vectors using Lipofectamine 2000 (Invitrogen). The cells were incubated at 37°C for 4 hr, the medium was replaced, and then the cells cultured for two more days. The dishes were harvested, YFP-positive cells sorted using flow cytometry, and the YFP-positive population purified further by selecting YFP-positive plaques in the presence of MPA for 2 rounds of purification. This produced VV strain VV-ΔTk- *yfp-gpt* with both loxP sites in the same orientation and VV strain VV-ΔTk-*yfp-gpt*-1loxP bearing a single loxP site.

Viruses bearing deletions of the *I4L* gene were produced using pDGloxPKO^INV^-Δ*I4L* and pDGloxPKO^DEL^-Δ*I4L* vectors in VV strain Western Reserve (WR), and in Δ*F4L* VV strains [23]. BSC-40 cells were infected for 1 hr (MOI = 2), transfected with plasmid DNA using Lipofectamine 2000, and virus harvested two days later. Recombinant virus were isolated using three rounds of plaque purification on BSC-40 cells in MPA selection media (DMEM supplemented with 10% serum, 50 U/mL penicillin, 50 µg/mL of streptomycin, 200 µM glutamine, 250 µg/mL xanthine, 15 µg/mL hypoxanthine, and 25 µg/mL MPA).

A recombinant ECTV was constructed by infecting BGMK cells with ECTV (strain Moscow) at an MOI = 0.01, followed by transfection with *Xho*I and *Not*I linearized pDGloxPKO-Δ*EVM002* DNA. The virus was harvested two days later and foci were purified twice in the presence of MPA and twice in the absence of MPA selection.

### Cell sorting and flow cytometry

U2OS cells were infected with virus at MOI ∼0.1 for 6 hr, and sorted for YFP fluorescence on a MoFlo cytometer (DakoCytomation). The top 5% of YFP positive cells (approximately 3,000 cells) were collected and mixed with ∼300,000 uninfected U2OS cells and cultured for two days in a 10 cm dish. For flow cytometry, ∼10^6^ HeLa cells were infected with virus at a MOI = 3 for 4 hr. The cells were suspended in 0.5 mL of PBS containing 1% FBS, and YFP fluorescence determined using a FACScan flow cytometer (Becton-Dickinson) and CellQuest software (Version 3.1).

### Luciferase and fluorescence detection using IVIS Imager

The fluorescence and bioluminescence were detected using a 200 Series IVIS Imager (Xenogen) and Living Image® v2.5 software. Cell lysates from Cre cell lines infected with VV-ΔTk*-yfp-gpt* were used to infect U2OS cells which were first imaged for YFP fluorescence prior to incubation with 20 µg of D-luciferin (Molecular Imaging Products Company) added directly to the cultured cells. D-luciferin was incubated with the cells for 20 min at 37°C. Identical program settings (exposure, aperture and pixel binning) were used for all experiments.

### Quantification of Cre recombination efficiency

U2OS cells stably expressing nuclear or cytoplasmic versions of U2OS, or U2OS cells transiently transfected with Cre recombinase were infected at an MOI of 0.05 for 2 days. Virus progeny was then analyzed by U2OS plaque assay for YFP expression of individual virus plaques.

### Fluorescence microscopy

To detect YFP, 2×10^5^ BGMK cells were seeded on coverslips and infected (or mock infected) with virus at a MOI = 0.01 for two days. The cells were fixed with 2% paraformaldehyde, mounted in 50% glycerol containing 4 mg/mL N-propyl-gallate (Sigma-Aldrich), and 250 µg/mL 4,6-diamidino-2-phenylindole (DAPI) (Invitrogen), and visualized using a Zeiss 710 confocal microscope equipped with Zeiss Zen software 2009, light edition. For immunofluorescence microscopy, 5×10^4^ Cre-expressing U2OS cells were plated per 1.8 cm^2^ well in Nunc Chamber Slides®. The cells were washed three times with PBS and fixed with 4% paraformaldehyde for 10 min at room temperature. The cells were treated for 30 min with blocking buffer (0.2% Triton X-100, 5% normal goat serum in PBS) and then incubated overnight at 4°C with a 1∶100 diluted anti-Cre antibody in blocking buffer. The cells were washed with PBS, incubated for 30 min with Cy3–conjugated goat anti-rabbit antibody (Jackson), washed three times, and then mounted on a slide. DNA was stained with 1.5 µg/mL 4,6-diamidino-2-phenylindole (DAPI). Cell images were captured using a Zeiss Axioplan 2 microscope equipped with Axioview 3.1 software.

### Virus DNA extraction

DNA was extracted from sucrose cushion purified virus stocks according to methods adapted from Meyer et al. [46]. Briefly, 200 µL of purified virus stock was treated with lysis buffer (1.4 mL 54% sucrose, 15 µL 2-mercaptoethanol, 50 µL if 20 mg/mL, proteinase-K and 250 µL 10% SDS) for 4 hours at 55°C. The samples were then purified by phenol extraction.

### Sequencing of viral DNA

Viral DNA from Cre-passaged viruses was subjected to PCR amplification at the site of Cre recombination. Sequencing was performed using the Applied Biosystem DNA analyzers. The entire genome of both parental VV Western Reserve and recombinant VV-Δ*I4L* ^DEL^ virus post Cre passage were sequenced from 5 µg of extracted viral DNA at the Genome Québec Innovation Centre (Montréal, Québec) using a high throughput pyrosequencing approach on a Roche 454 GS FLX Titanium sequencer platform. CLC Genomics Workbench 4.6 software was used to assemble the genome sequence. The viral genomes were analyzed and compared using dotplots prepared using the program Gepard [47] with default settings.

### DNA restriction digest and southern hybridization

Viral DNA was analyzed using Southern blot. Briefly, 7 µg of DNA was digested overnight with HindIII restriction enzyme (Invitrogen) at 37°C. DNA digests were electrophoresed on a 0.8% agarose gel in 1× TAE buffer at 46 V for 6 hours, then stained with ethidium bromide and photographed. Gels were first denatured for 45 mins, then neutralized for 45 mins prior to transfer over night onto Hybond-N membrane (Amersham), and finally UV cross-linked. A 931 bp *yfp-gpt* probe was labeled with P^32^ α-dCTP (PerkinElmer) using the Multiprime DNA Labeling kit (Amersham). The blot was blocked for 2 hours at 42°C in a formamide pre-hybridization buffer followed by over night hybridization with 5 µCurries of denatured probe. Membranes were washed and exposed to a phospho screen for 18 hours and imaged on a Storm imaging system (Storm 860; Molecular Dynamics) using ImageQuant software, version 5.2.

### Western blotting

For immunoblotting, cells were lysed in RIPA buffer with protease inhibitors (Complete®, Roche) for 30 min on ice. The cell lysates were sonicated for 30 seconds, centrifuged at 10,000×*g* and the cell pellets discarded, fractionated by electrophoresis on a Nupage 4–12% Bis-Tris gel (Invitrogen), transferred to Hybond-C extra nitrocellulose membranes (Amersham), and probed with the indicated primary antibodies. Secondary antibodies conjugated to horseradish peroxidase or Li-Cor infrared dyes were used to detect bound antigens.

### Statistical Analysis

Data were analyzed by a one-way Anova, followed by a Turkey post-hoc for multiple group comparison at a 99% confidence interval.

## Supporting Information

Figure S1 **Detection of YFP protein expression by western blot analysis.** (**A**) Western blotting of transfected cells. Anti-YFP western blot of U2OS cells mock transfected (1), or transfected with plasmid DNA pEYFP-c1 (2), pEYFP-gpt, (3), or pEYFP-gpt-1loxP (4).
(TIFF)

Figure S2 **Plasmid maps of pDGloxPKO^DEL^ and pDGloxPKO^INV^.** (**A**) Map of cloning vector pDGloxPKO^DEL^ and (**B**) pDGloxPKO^INV^ with labeled open reading frames.
(TIFF)

Figure S3 **Sequencing analysis of genomic viral DNA from VV-Δ** ***I4L*** **^DEL^.** (**A**) Dotplot comparison of sequenced viral genomes of wild type Western Reserve vaccinia virus and recombinant VV-Δ*I4L* ^DEL^ virus post Cre passage using default settings available in Gepard [47].
(TIFF)

Table S1 **Primers used in the study.**
(DOC)
