# Supplementary figures and images for: A Selectable and Excisable Marker System for the Rapid Creation of Recombinant Poxviruses
Source: PLoS One. 2011 Sep 8;6(9):e24643. doi: 10.1371/journal.pone.0024643 (PMC3169633; doi:10.1371/journal.pone.0024643)

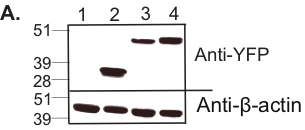

Supplement: Figure S1 — Detection of YFP protein expression by western blot analysis. (A) Western blotting of transfected cells. Anti-YFP western blot of U2OS cells mock transfected (1), or transfected with plasmid DNA pEYFP-c1 (2), pEYFP-gpt, (3), or pEYFP-gpt-1loxP (4). (TIFF) [file pone.0024643.s001.tiff]

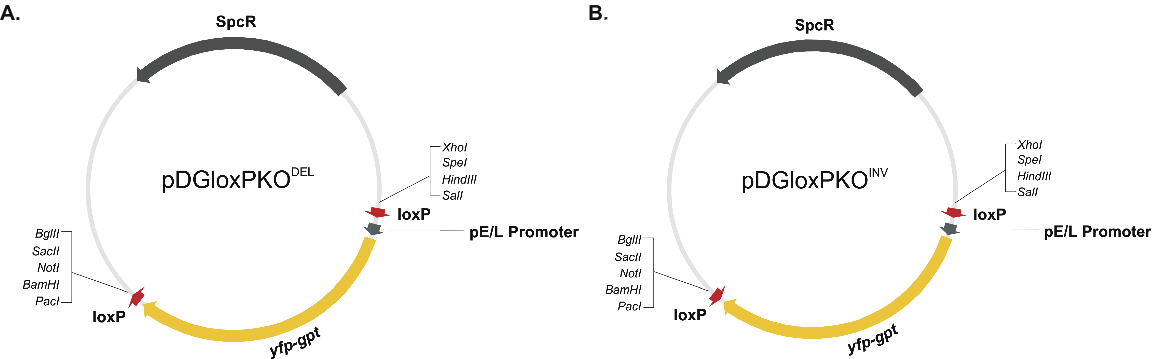

Supplement: Figure S2 — Plasmid maps of pDGloxPKODEL and pDGloxPKOINV. (A) Map of cloning vector pDGloxPKODEL and (B) pDGloxPKOINV with labeled open reading frames. (TIFF) [file pone.0024643.s002.tiff]

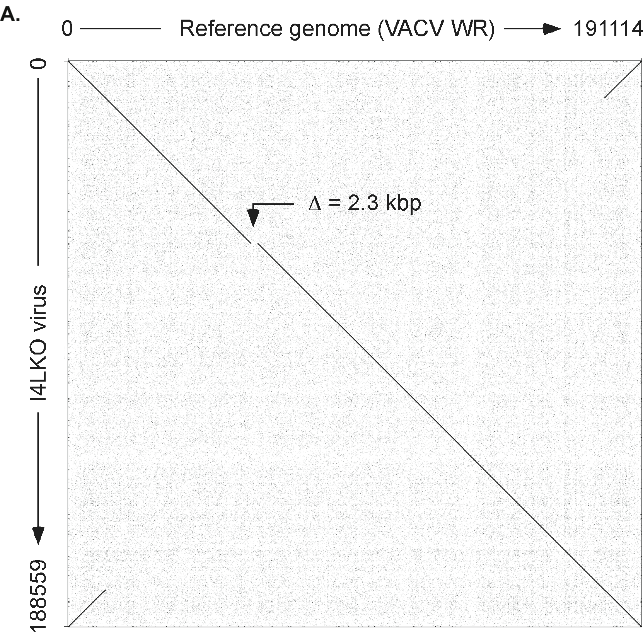

Supplement: Figure S3 — Sequencing analysis of genomic viral DNA from VV-Δ I4L DEL. (A) Dotplot comparison of sequenced viral genomes of wild type Western Reserve vaccinia virus and recombinant VV-ΔI4L DEL virus post Cre passage using default settings available in Gepard [47]. (TIFF) [file pone.0024643.s003.tiff]
