# Supplementary material for: A Selectable and Excisable Marker System for the Rapid Creation of Recombinant Poxviruses
Source: PLoS One. 2011 Sep 8;6(9):e24643. doi: 10.1371/journal.pone.0024643 (PMC3169633; doi:10.1371/journal.pone.0024643)
Supplement: Table S1 — Primers used in the study. (DOC) [file pone.0024643.s004.doc]

**Supporting Information**

**Table S1**. List of primer sequences used in this study

| **Primer Name** | **Purpose** | **Sequence (5' to 3')** |
| --- | --- | --- |
| loxP | Creation of loxP sites | GTCAGATCCGCTAGCTCGAGATAACTTCGTATAATGTATGCTATACGAAGTTATCTAGCGCTACCGGTC |
| I5L -left | Creation of pDGloxPKO-Δ*I4L* vector | ACTAGTGGAAGGGTATCTATACTTATAGAATAATC |
| I5L -right |  | GTCGACTTTTGTTGGTGTAATAAAAAAATTATTTAAC |
| I3L -left |  | CCGCGGGGTTAAACAAAAACATTTTTATTCTC |
| I3L -right |  | AGATCTGTTTAGTCTCTCCTTCCAAC |
| EVM002-5' -left | Creation of pDGloxPKO^DEL^-ΔEVM002 vector | AAGCTTCTCATAATGATTTACTTTTTC |
| EVM002-5' -right |  | CTCGAGCGATTCCGTCCAAGATGATAA |
| EVM002-3' -left |  | GCGGCCGCGGTGCTATATCTTTTCCGTTT |
| EVM002-3' -right |  | GGATCCTAGAAAGAAAATATTTAAAAA |
| NLS-free Cre -left | Creation of Cre plasmid pLPCX-Cyto_Cre | CCTCGAGGATGTCCAATTTACTGACCGTACACC |
| NLS-free Cre -right |  | GCGGCCGCCTAATCGCCATCTTCCAGCAGGCGC |
